# Maximizing the Value of Drug Stockpiles for Pandemic Influenza

**DOI:** 10.3201/eid1510.090844

**Published:** 2009-10

**Authors:** Alain Li Wan Po, Peter Farndon, Nick Palmer

**Affiliations:** National Genetics Education and Development Centre, Birmingham, UK (A. Li Wan Po, P. Farndon); House of Parliament, London, UK (N. Palmer).

**Keywords:** Pandemic, influenza, drug stockpiles, preparedness, oseltamizir, Tamiflu, letter

**To the Editor:** Tamiflu (oseltamivir; Roche, Indianapolis, IN, USA) is destined to be one of the few branded drugs to develop instant street recognition because of its status as 1 of only 2 licensed drugs shown to be active against the influenza A pandemic (H1N1) 2009 virus. Tamiflu is the major drug stockpiled by governments around the world in preparedness against an influenza pandemic. More than 70 governments have placed orders for Tamiflu, and at least 220 million treatment courses have been stockpiled since 2003 at a cost of $6.9 billion (*1*). Roche is producing 110 million courses for the 5 months from May to fall 2009 and will produce up to 36 million courses per month by year’s end if necessary. Given the estimated world population of 6.8 billion, it is clear that, on a global basis, stockpiles are woefully inadequate. For the United Kingdom, official estimates indicate sufficient stocks currently exist for half of the population (*2*).

Given the high cost of these stockpiles, every effort should be made to maximize usage of the drug. Most of us are aware of shelf-life assignment to foods, a concept first applied to drugs well before its adoption by food manufacturers. Shelf-life extension could potentially yield significant cost savings in the event stockpiled drugs are not required for use within the typical 5-year shelf life. We use Tamiflu as a case example to suggest how this could be done through careful evidence-based risk assessment.

The chemical integrity of any medicine, including Tamiflu, is important because decomposition may lead to loss of activity or formation of toxic products. For formulated products, decomposition may lead to impaired bioavailability. As it became obvious that there were inadequate drug stockpiles even in affluent countries, one of our authors (A.L.W.P., who had served as a member of the UK Committee on Safety of Medicines [*3**,**4*]) wrote to his local member of parliament (coauthor N.P.) to suggest that the government institute a program to extend the shelf life of drug stockpiles. A.L.W.P. argued that the relatively minor development work necessary to implement a shelf-life extension program would be highly cost-effective. The UK Department of Health then initiated a collaboration with Roche to extend the shelf life of Tamiflu. On May 8, 2009, the European Medicines Agency independently advised that new batches of Tamiflu would have a shelf life of 7 years instead of only 5 years (*5*).

Oseltamivir is a prodrug that needs metabolic activation (Figure) (*6*). Prodrugs are used typically to reduce toxicity caused by functional groups such as the carboxylate ion, to alter release properties (e.g., prolonging action of antipsychotic agents), or to improve absorption (bioavailability) by making the drug more lipophilic.

**Figure:**
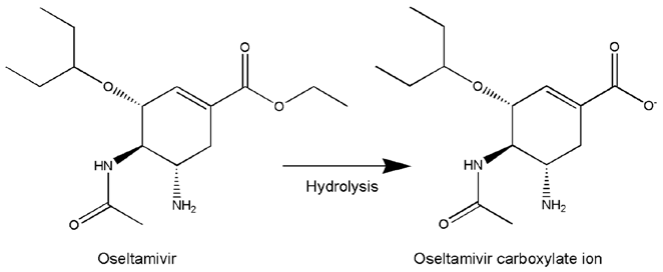
Metabolic activation of oseltamivir to carboxylic acid.

Oseltamivir carboxylic acid has poor bioavailability; <5% orally compared to 80% for oseltamivir, the parent drug (*7*). The carboxylate is the only major metabolite and the principal degradation product (*7*,*8*). Therefore, with poor storage the major risk is reduced activity through reduced absorption rather than formation of toxic by-products.

Health agencies also stockpile other drugs (*9*), most notably antimicrobial drugs and vaccines. Shelf-life extension would need to be assessed on a product-by-product basis. For example, antimicrobial drugs are often quite unstable and the toxicologic implications are less clear; some evidence suggests that allergenic polymers could be formed while the drugs are in storage. On the positive side, antimicrobial drugs are considerably less expensive than neuramidase inhibitors such as Tamiflu, making antimicrobial stockpiles less costly to replenish.

Products with more complicated delivery systems, such as zanamivir in inhalers, would require more validation. For biological products in complex formulations such as vaccines, stability validation may not be cost-effective.

Given the high costs involved in maintaining adequate drug stockpiles, attempts should be made to optimize the value of drugs; shelf-life extension is one of the easiest and most cost-effective ways of doing this. We suggest that governments undertake a systematic program for iterative shelf-life extension, ideally cooperatively. The considerable financial savings could mitigate drug shortages of expensive antiviral drugs. The chemical profile of oseltamivir and its degradation pathway suggest that extending the shelf life of Tamiflu to >20 years should be feasible. Storage in dry airtight containers should be able to maintain the integrity of the product for >7 years. During a pandemic, when supplies are unavailable, the balance of benefit to harm would favor using the expired product.

The 1918 influenza pandemic is estimated to have killed 50 million persons worldwide (*10*), many in developing countries. By better safeguarding available drug stockpiles, more drugs could be made available to poorer countries that have few drugs stockpiled.
